# M^1^A and m^7^G modification-related genes are potential biomarkers for survival prognosis and for deciphering the tumor immune microenvironment in esophageal squamous cell carcinoma

**DOI:** 10.1007/s12672-023-00710-6

**Published:** 2023-06-14

**Authors:** Ruixi Wang, Xingyuan Cheng, Dongmei Chi, Shiliang Liu, Qiaoqiao Li, Baoqing Chen, Mian Xi

**Affiliations:** 1 State Key Laboratory of Oncology in South China, Collaborative Innovation Centre for Cancer Medicine, Guangdong Esophageal Cancer Institute, Guangzhou, China; 2 Department of Radiation Oncology, Sun Yat-Sen University Cancer Center, No. 651 Dongfeng East Road, Guangzhou, 510060 China; 3 Department of Anesthesiology, Sun Yat-Sen University Cancer Center, No. 651 Dongfeng East Road, Guangzhou, 510060 China

**Keywords:** Methyladenosine, Esophageal squamous cell carcinoma, Prognostic biomarker, Treatment response, Immune cell infiltration

## Abstract

**Background:**

Esophageal squamous cell carcinoma (ESCC) is the most common esophageal malignancy, and RNA methylation has been reported to be involved in the tumorigenesis of ESCC. However, no study has explored methylation modifications in m^1^A and m^7^G as prognostic markers for survival prediction in ESCC.

**Methods:**

Public gene-expression data and clinical annotation of 254 patients obtained from The Cancer Genome Atlas and the Gene Expression Omnibus databases were analyzed to identify potential consensus clusters of m^1^A and m^7^G modification-related genes. The RNA-seq of 20 patients in Sun Yat-Sen University Cancer Center was used as the validation set. Following screening for relevant differentially expressed genes (DEGs) and enrichment pathways were elucidated. DEGs were used to construct risk models using the randomForest algorithm, and the prognostic role of the models was assessed by applying Kaplan–Meier analysis. Extent of immune cell infiltration, drug resistance, and response to cancer treatment among different clusters and risk groups were also evaluated.

**Results:**

Consensus clustering analysis based on m^1^A and m^7^G modification patterns revealed three potential clusters. In total, 212 RNA methylation-related DEGs were identified. The methylation-associated signature consisting of 6 genes was then constructed to calculate methylation-related score (MRScore) and patients were dived into MRScore-high and MRScore-low groups. This signature has satisfied prognostic value for survival of ESCC (AUC = 0.66, 0.67, 0.64 for 2-, 3-, 4- year OS), and has satisfied performance in the validation SYSUCC cohort (AUC = 0.66 for 2- and 3-year OS). Significant correlation between m^1^A and m^7^G modification-related genes and immune cell infiltration, and drug resistance was also observed.

**Conclusions:**

Transcriptomic prognostic signatures based on m^1^A and m^7^G modification-related genes are closely associated with immune cell infiltration in ESCC patients and have important correlations with the therapeutic sensitivity of multiple chemotherapeutic agents.

**Supplementary Information:**

The online version contains supplementary material available at 10.1007/s12672-023-00710-6.

## Introduction

Esophageal cancer is the seventh most common cancer and the sixth leading cause of cancer death worldwide [1]. Esophageal squamous cell carcinoma (ESCC) and esophageal adenocarcinoma (EAC) are the most prevalent histological subtypes of esophageal cancer, of which ESCC accounts for more than 90% of all patients with esophageal cancer [2]. China is one of the countries with a high incidence of esophageal cancer. Although studies have shown that the 5-year survival rate of esophageal cancer has increased from 20.9% to 30.3%, it remains a leading burden on the health system and an important cause for a shorter life expectancy in the last decade [3, 4]. Age, gender, occupation, race, geography, living environment, dietary habits, and genetic susceptibility are the population risk factors for the development of esophageal cancer, whose occurrence and progression are a multi-stage, multi-factorial process involving complex interactions between the genome, transcriptome, and epigenome [5]. Therefore, in-depth studies on the predisposing factors and potential tumorigenic mechanisms underlying the occurrence and progression of esophageal cancer, and the discovery of diagnostic and prognostic biomarkers are urgently needed.

With a worldwide overall 5-year survival rate of only 30% for patients with esophageal cancer and less than 15% for patients with advanced disease, early diagnosis, and accurate prognosis are key to improving survival and refining therapy [6–8]. However, due to the heterogeneity of cancers at the molecular level, clinical outcomes and prognosis vary despite patients being in the same stage and receiving similar treatments [9]. Consequently, the significance of molecular indicators in the early detection, therapeutic surveillance, and evaluation of prognosis in esophageal cancer has become increasingly paramount.

RNA methylation plays a crucial role in the development of cancer [10, 11], particularly by regulating adaptive immunity in cancer and altered methylation status, which can affect the responsiveness of patients with solid tumors to immune checkpoint inhibitor (ICI) therapy [12]. With the development of RNA sequencing technology, many RNA modifications, including 5-methylcytosine, N1-methyladenosine, N6-methyladenosine, N7-methyladenosine etc., were discovered continuously [13, 14]. Alterations in the methylation status within the hypoxic environment of the tumor promote the development of metastasis [15]. The above studies suggest that RNA methylation may play a role in the tumorigenesis and the tumor microenvironment of ESCC [16, 17]. N7-methylguanosine (m^7^G) is a methylation modification that occurs most frequently in transfer RNA (tRNA) and contributes to the maintenance of tRNA stability, as well as other types of RNAs [18, 19]. Among hundreds of known RNA modifications, m^1^A modification is one of the most common internal modifications in messenger RNA (mRNA), affecting RNA shearing, translation, stability and regulatory effects of certain non-coding RNAs. On average, there are one to two m^1^A sites per 1,000 RNA nucleotides in mammalian cells. The aberrant expression of m^1^A regulators in a variety of tumors affects the regulation of malignant biological behaviors such as cell proliferation, invasion and metastasis [20, 21]. The prognostic role of m^1^G and m^7^G modification in esophageal cancer, and their biological function in this malignancy have not been reported, warranting the need for this study.

To achieve this goal, we first constructed a transcriptomic risk model for m^7^G and m^1^A based on available public data and then evaluated its relationship to the clinicopathological characteristics and prognosis of ESCC patients. We explored the effects of the risk model on immune infiltration, immune cell abundance, and chemotherapeutic drug sensitivity, thereby aiming to furnish personalized therapeutic strategies and clinical prognostic survival oversight for patients suffering from ESCC.

## Materials and methods

### Selection of m^1^A and m^7^G modification-related genes and acquisition of data from public repositories

Figure S1 shows the study workflow. As reported in previously published researches, 7 m^1^A (*ALKBH1, ALKBH3, FTO, TRMT10C, TRMT6, TRMT61A, YTHDF1, YTHDF2*) and 16 m^7^G (*AGO2, CYFIP1, EIF3D, EIF4E2, EIF4E3, EIF4G3, GEMIN5, IFIT5, LARP1, METTL1, NCBP1, NCBP2, NCBP3, NUDT11, SNUPN*) regulator-mediated modification patterns (Supplementary Table 1) were identified for further analyses from published literature [22–26].

Counts RNA-seq and clinical information for TCGA-ESCC were acquired through the UCSC Xena Browser (https://xenabrowser.net/) [27]. Also, one eligible GSE53625 cohort with prognostic data and single-cell RNA-seq (scRNA) data from the GSE188900 dataset were acquired from the Gene Expression Omnibus (GEO) database (https://www.ncbinlmnihgov/) [28, 29]. RNA-seq data for TCGA and GEO were corrected for batch effects by the “ComBat” algorithm to uniformly rearrange and improve compatibility, and eventually translated into TPM data [30]. For each patient included, we retrieved clinical data containing age, gender, tumor stage, TNM staging, and survival status. RNA-seq data of 20 patients with ESCC from the Sun Yat-Sen University Cancer Center compose an independent SYSUCC cohort, including information on each patient's response to chemotherapy.

ESCC patients who met the following criteria were included in this analysis: (1) ESCC patients with at least 30 days of follow-up; (2) primary esophageal tumor; (3) available mRNA, lncRNA and miRNA gene expression levels for analysis; (4) basic demographic information; (5) detailed pathological information was optional. Exclusion criteria were as followed: (1) secondary tumors in the esophagus; (2) concurrent primary tumors at other sites. Finally, 254 ESCC cases comprising 77 TCGA and 177 GSE53625 samples with complete disease profiles and RNA expression data were included for prognosis analysis [27, 28], and 20 ESCC patients from the SYSUCC cohort formed an independent validation set (Supplementary Table 2).

### Identification of potential gene markers for m^1^A and m^7^G modification and molecular subtype stratification

Consensus clustering analysis was performed on transcriptomic data obtained from 254 ESCC patients using a K value of the consensus matrix set to 3, to obtain the RNA methylation modification status and potential prognosis-related genes. The clustering analysis was performed using the R package "ConsensesclusterPlus" (version 1.60.0) with the main parameters of 500 resamples, 0.8 sampling rate, maxK = 6, "pam" clustering algorithm and Euclidean distance algorithm [31]. We utilized the R packages "pca3d" and "Rtsne" to classify the three clusters that emerged from our consensus clustering analysis. The stability of these subtypes was visually evaluated and confirmed using the cumulative distribution function (CDF) curve.

Differentially expressed genes (DEGs) between ESCC tissues were screened using the "limma" package in R software, with the selection criteria of |log2 (FoldChange)|> 0.585 and adjusted *P*-value < 0.05 [32]. After a two-by-two comparison, the screened DEGs from the union set were plotted by the Venn diagram. Univariate Cox regression analysis included all the selected DEGs was performed to determine their prognostic significance, and indicators with *P*-value < 0.05 were earmarked for further investigation.

### Signaling pathway enrichment analysis of m^1^A and m^7^G modification-associated DEGs

Gene ontology (GO) functional enrichment analysis and Kyoto Encyclopedia of Genes and Genomes (KEGG) metabolic pathway enrichment analysis of the DEGs and core genes were performed using the R software packages clusterProfiler, org.Hs.eg.db, DOSE, enrichplot, colorspace, etc. [33, 34]. The GO category enrichment analysis included biological process (BP), cellular component (CC), and molecular function (MF). GO and KEGG functional enrichments were identified via hypergeometric tests with FDR corrections.

Fisher's exact test was used in the enrichment analysis to test whether the DEGs were enriched in a network and a corrected *P*-value < 0.05 was set as the screening condition. Histograms were plotted using ggplot2 software package in R software. All DEGs were subjected to Gene Set Enrichment Analysis (GSEA) and Gene Set Variation Analysis (GSVA) analyses, with the majority of the enriched pathways obtained by GSEA analysis being connected to immunity. GSVA analysis was performed with "h.all.v7.5.1.symbols.gmt " using R package "GSVA" [35, 36].

### Construction and efficacy evaluation of m^1^A and m^7^G modification-related transcriptome prognostic tool

After analyzing the prognostic significance of the screening data using the Random Forest algorithm of the R-language randomForest software package, key prognostic m^1^A and m^7^G modification-related genes were extracted. The relative importance was then screened to select the important genes to construct the risk score model. Based on the expression of the genes included in the model, patients were divided into MRScore-high and MRScore-low groups by median MRScore (Methylation-related score).The MRScore was calculated as the sum of the products of gene expression levels and coefficients: $MRScore={\sum }_{i=1}^{n}{Coef}_{i}\times {Exp}_{i}$. Subsequently, Kaplan–Meier analysis and receiver operating characteristic (ROC) curves were applied to assess the prognostic value of the MRScore model. In addition, we performed an external validation basing the SYSUCC cohort, and tested the predictive value of MRScore to the efficacy of chemotherapy in patients with ESCC. To further enrich the clinical connotation of the model, we established the nomogram model by integrating the MRScore and clinical characteristics, and tested the goodness of the fit of the model using the Hosmer-leme test.

### Analysis of immune cell infiltration and drug sensitivity-related functions based on the prognostic markers of m^1^A and m^7^G modification

To determine whether RNA methylation is correlated to altered immune cell levels and immune pathways in ESCC patients, we evaluated immune infiltration level utilizing two distinct algorithms, CIBERSORT and ssGSEA [37, 38]. CIBERSORT uses a deconvolution algorithm to assess the composition and abundance of immune cells based on the expression matrix of mixed cells. Then, ssGSEA algorithm was used to calculate the enrichment scores of single samples and immune cell marker gene set pairs to determine the extent of immune infiltration. The R package "randomForest" was used to display a correlation heat map based on multivariate regression to predict the importance and composition of key genes, indicating the potential immunological contribution of prognostic model genes, and identifying the main predictors. The TIDE algorithm predicts the efficacy of immune checkpoint blockade (ICB) therapy in ESCC patients based on T-cell dysfunction and T-cell rejection analysis features in immunosuppression [39].

ScRNA-seq data in the form of RNA-Seq Expectation Maximization (RSEM) software standardized counts was retrieved from the GSE188900 dataset within the GEO database. The dataset included 4 patients with esophageal squamous carcinoma [29]. ScRNA-seq data was analyzed by using the R packages "Seurat" and "harmony". Cells with more than 5% of mitochondrial genes or less than 200 genes, and data with cell counts less than 3 were removed [40]. We performed principal component analysis (PCA) using the first 1500 variable genes in the dataset to identify marker genes for different cell types and showed descending subgroups in t-distributed stochastic neighbor embedding (t-SNE) plots to assign the MRScore of each tumor cell sample according to the AddModuleScore function.

### Stemness index mRNAsi analysis and drug sensitivity analysis based on the prognostic model

To improve the practical clinical value of the model and to further analyze the functional role of methylation-related RNAs on therapeutics, we tested the effect of m^1^A and m^7^G modifications on sensitivity to treatment with multiple novel chemotherapeutic agents. The Optimal Classification with Logistic Regression (OCLR) machine learning methodology was used to ascertain the ‘stemness index’, or mRNAsi, quantifying the degree of resemblance between neoplastic and stem cells. The Connectivity Map database (CMap) was used to identify potential drugs or compounds based on mRNAsi pattern [41, 42].

Drug sensitivity was predicted by the R package "pRRophetic" based on the Cancer Genome Project (CGP) database (https://www.sanger.ac.uk/science/programmes/cancer-genome-project). To enrich the predictive clinical value of the model, prediction was performed by the R package "oncoPredict" based on the Genomics of Drug Sensitivity in Cancer (GDSC) database (https://www.cancerrxgene.org/) [44–46].

### Statistical analysis

All statistical analysis was performed using R software (version 4.2.0, Vienna, Austria). The Wilcoxon-Mann–Whitney U test was used to analyze the differences between the two data sets. Survival analyses were performed using log-rank test of the Kaplan–Meier survival curves to assess the effect of the high- and low- expression of risk model on the OS of patients. The limma package was used to analyze the differential genes in the samples of three clusters of the m^1^A and m^7^G modification-associated genes. Differences were statistically significant when *P*-value < 0.05, and the False Discovery Rate (FDR) approach for nuanced adjustments to control for multiple comparisons.

## Results

### Correlation of the m^1^A and m^7^G regulators in esophageal cancer

**Fig. 1:**
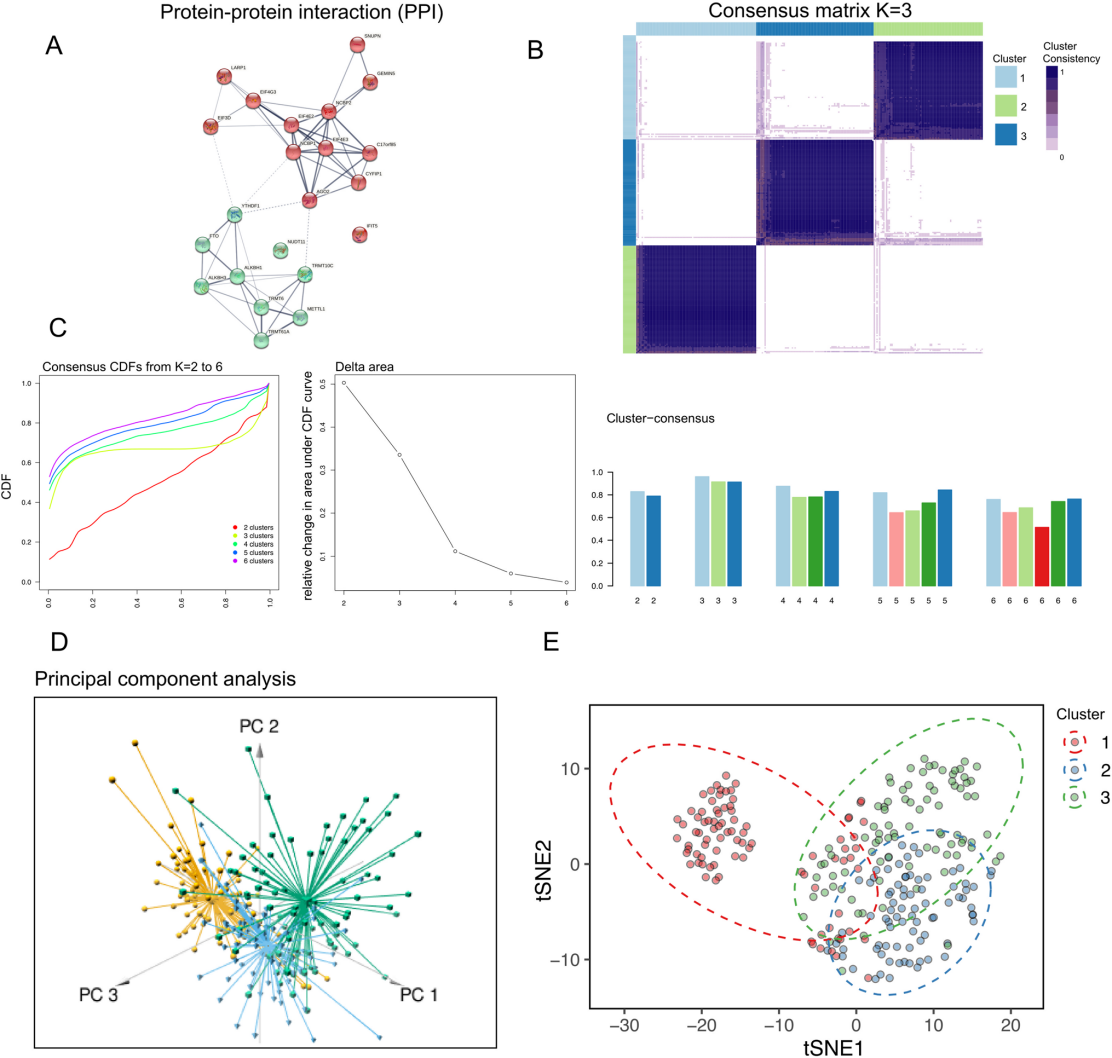
Consensus clustering analysis and preliminary functional modality evaluation of genes associated with m^1^A and m^7^G modification expression in ESCC. **A** PPI protein network clustering analysis was performed on 23 m^1^A and m^7^G modification-related genes. Among them, most of the m1A- related genes were clustered into one category, and m7G- related genes into another. **B**, **C** Display graph of formula clustering analysis of m^1^A and m^7^G modification-related genes. K value of consensus matrix is set at 3. Inter-cluster distribution of obtained subsets for different K values in consensus clustering analysis. Occurrence of relative change in area under CDF curve when K value of consensus matrix is set to 2, 3, 4, 5, 6. **D**, **E** PCA and t-SNE plot visualization results of the three subsets identified by consensus clustering analysis

The schematic representation delineating the sequential steps of our analysis is comprehensively illustrated in Figure S1. A technical normalization and description of the outcomes both before and after the correction of the batch effect (Figure S2A) were shown. Stratification of the m^1^A and m^7^G modification-associated genes in the protein–protein interaction (PPI) enrichment network showed a close association and high correlation among the following m^7^G-related genes, *SNUPN*, *LARP1*, *EIF4G3*, *EIF3D*, *GEMINS*, *NCBP2*, *NCBP1*, *AGO2*, and *CYFIP1* (red spheres). Most m^1^A-related genes also possessed high correlation (green spheres) (Fig. 1A). Figure S2B illustrates the distribution of 23 m^1^A and m^7^G-related genes on the chromosome. Correlations between the well-known m^7^G regulator, METTL1, and the m^1^A regulator, NCBP3 (Figure S2C) was observed.

### Identification of sub-clusters and their DEGs based on m^1^A and m^7^G regulators

**Fig. 2:**
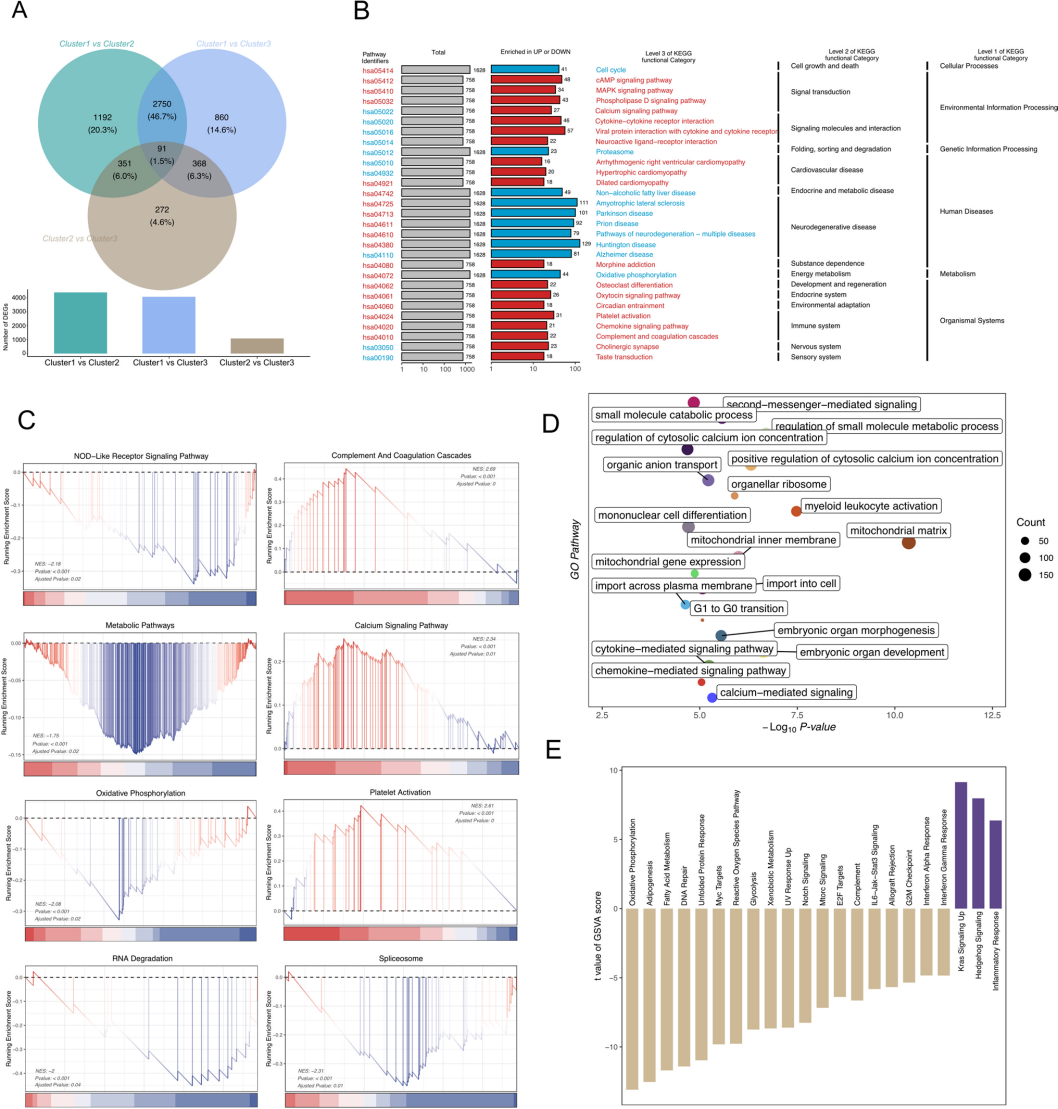
m^1^A and m^7^G modification-related gene clustering subset pathway enrichment analysis. **A** The union of three screened DEGs set. **B** The resultant state of KEGG analysis of the three subsets obtained by consensus clustering. **C** GSEA of the functionally linked relevance of several key pathways to RNA methylation modifications. **D** The resultant state of GO enrichment analysis of differential expression genes. **E** GSVA assessed the subset of genes associated with m^1^A and m^7^G modification expression functional linkage

Then, we identified the correlation patterns of potential gene markers for m^1^A and m7G modification by consensus clustering analysis, and the K value of the consensus matrix was set to 3. The occurrence of relative changes in the area under the CDF curve when the K value of the consensus matrix was set to 2, 3, 4, 5, and 6 were compared (Fig. 1B, C, Figure S2D). Finally, the CDF curve identified three stable subtypes, and the distribution and regional positions were captured (Fig. 1D, E). Exploration of the DEGs in the three potential clusters led to the identification of 5884 genes. Figure 2A illustrates the Venn map for screening DEGs.

### Functional enrichment analyses of three clusters-related DEGs

KEGG analysis of the DEGs in the three clusters stratified by m^1^A and m^7^G modification-related genes showed that different KEGG functional categories were highly enriched (*P*-value < 0.05) at three levels (Supplementary Table S3). KEGG pathways that were significantly enriched in upregulated genes are shown in red; those significantly enriched in genes downregulated are shown in blue (Fig. 2B). The gene pathway, hsa05414, which is functionally related to cell cycle and associated with cellular processes, was enriched with downregulated expression of 41 genes. The hsa05412 pathway, which is closely associated with cAMP signaling pathways, was enriched with 48 upregulated genes. The GSEA-KEGG analysis of DEGs among the three subgroups of ESCC demonstrated that the three cluster subsets (Supplementary Table S4) are connected to the upregulation of complement and coagulation cascades, calcium signaling pathway, and the platelet activation pathway, and downregulation of NOD-like receptor signaling pathway, etc. (Fig. 2C). The GO top 20 pathway shows enriched genes, with larger circles representing more genes (Fig. 2D). The outcomes of the GSVA analysis, featuring hallmark gene sets manifesting substantial enrichment, are displayed in Fig. 2E.

### Prognostic risk model construction and preliminary assessment of esophageal cancer based onm^1^A and m^7^G modification cluster-related DEGs

**Fig. 3:**
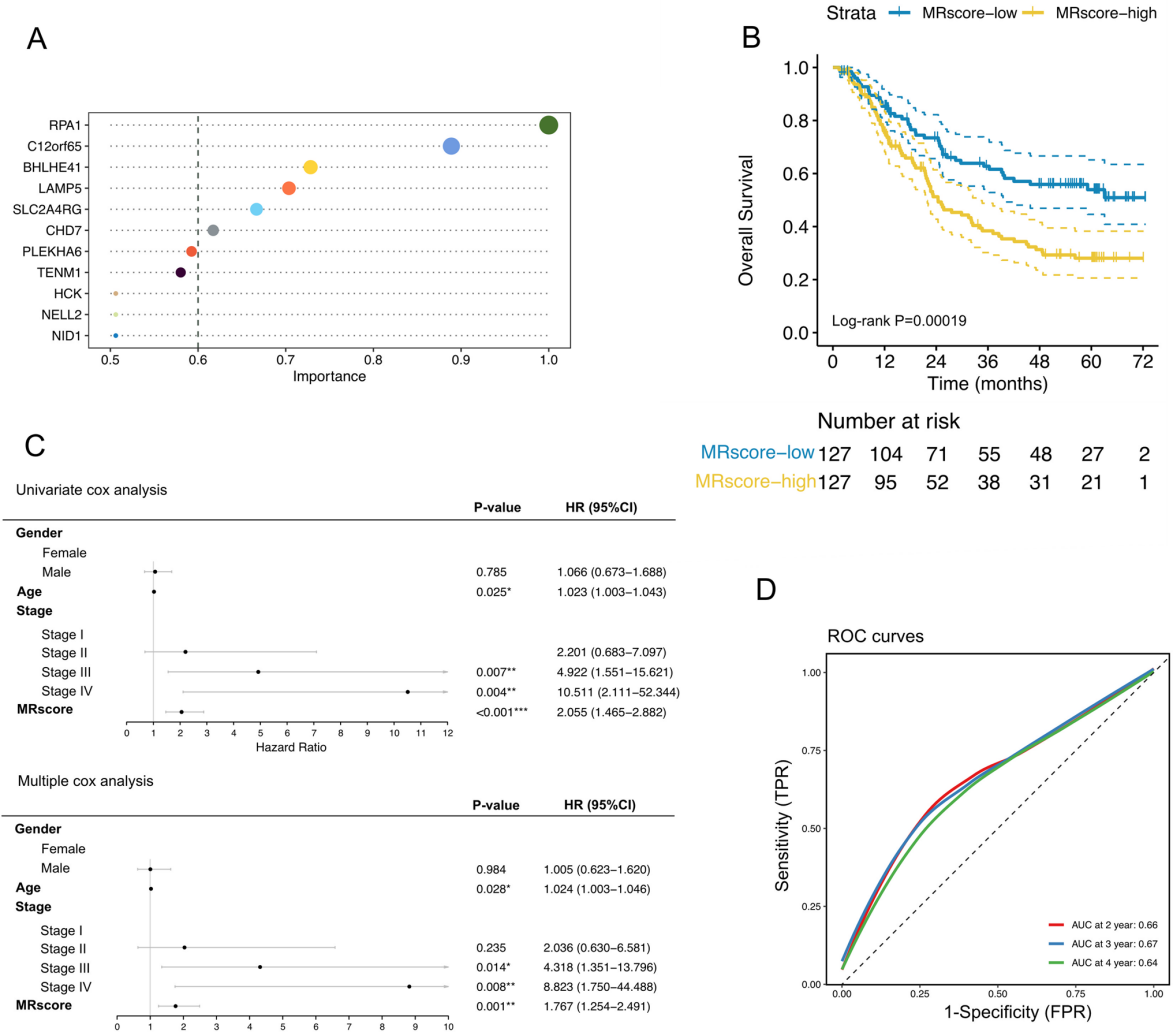
Prognostic risk model construction and preliminary assessment of esophageal cancer based on m^1^A and m^7^G modification-related genes. **A** Screening of 6 key genes by randomForest algorithm.** B** Comparison of overall survival curves for MRScore-low and MRScore-high patients. **C** Forest plots of the univariate Cox regression and multivariate Cox regression regarding OS. **D** ROC curves of prognostic risk model

The randomForest algorithm (Fig. 3A) applied to 212 RNA methylation-related DEGs narrowed the screen to 6 key genes that relative importance > 0.6 were used to construct methylation-related score (MRScore) models (Supplementary Table 5), MRScore was calculated as the formula: 0.0965*BHLHE41 + 0.3004*C12orf65 − 0.2942*CHD7 − 0.0003*LAMP5 + 0.0856*RPA1 + 0.0860*SLC2A4RG. Patients were categorized into MRScore-high and MRScore-low groups based on the median MRScore (methylation-related score). Kaplan–Meier curves demonstrated that the methylation modification risk model we constructed could significantly differentiate the survival of ESCC patients (Fig. 3B). Univariate and multivariate cox regression analysis of OS showed that the MRScore, age, and clinical III-IV stage were independent prognostic factors (Fig. 3C). The areas under the curve (AUC) values of the ROC curves for 2-, 3-, and 4- years OS, were 0.66, 0.67, and 0.64 respectively (Fig. 3D).

**Fig. 4:**
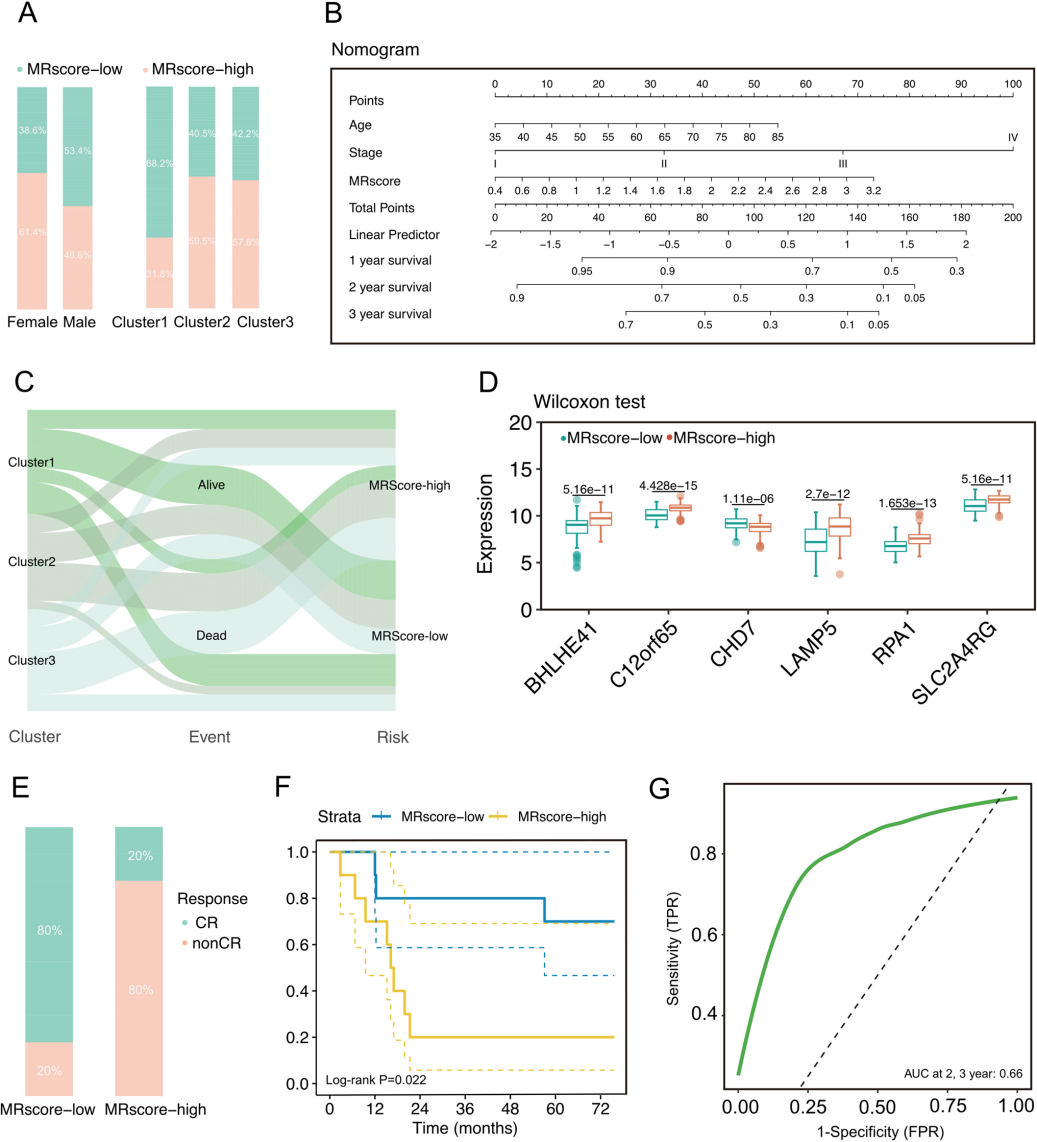
Correlation between clinical data and prognostic models. **A** Histogram of the distribution of clinical base information of patients in the MRScore-low and MRScore-high groups. **B** Nomogram combining the composition of MRScore, risk factor age, and stage. **C** Sankey plot showing the clinical transitions towards the constructed predictive risk model. **D** Box plots of expression distribution of 6 key RNA methylation- associated genes. **E** Bar diagram presents the distinctions of treatment outcomes between the MRScore-low and MRScore-high groups. **F** Kaplan–Meier curves for the independent external cohort. **G** The ROC curves for the SYSUCC cohort at 2- and 3- year OS

The distribution of clinical underlying information of patients in the MRScore-low and MRScore-high groups was showed in Fig. 4A. The nomogram model was established by integrating the risk score, and clinical characteristics including age and stage (Fig. 4B) showed a high goodness of fit (Figure S2E), as shown by the Hosmer-leme test. Sankey plot was used to further evaluate the direction of the prognostic risk model for ESCC patients in the forementioned three clusters (Fig. 4C). Figure 4D shows the difference expression of 6 hub genes in the MRScore-low and MRScore-high groups, with *P*-value corrected by the 'FDR' method. The inclusion of 20 patients from the SYSUCC cohort for independent external validation showed that the model was stable. Additionally, it has been proven that MRS can hemotherapy of patients with ESCC (Fig. 4E, F). The ROC curves for the SYSUCC cohort at 2- and 3- year OS had an AUC of 0.66. (Fig. 4G).

### Immune infiltration analysis of the prognostic model of ESCC based on m^1^A and m^7^G modification cluster-associated genes

**Fig. 5:**
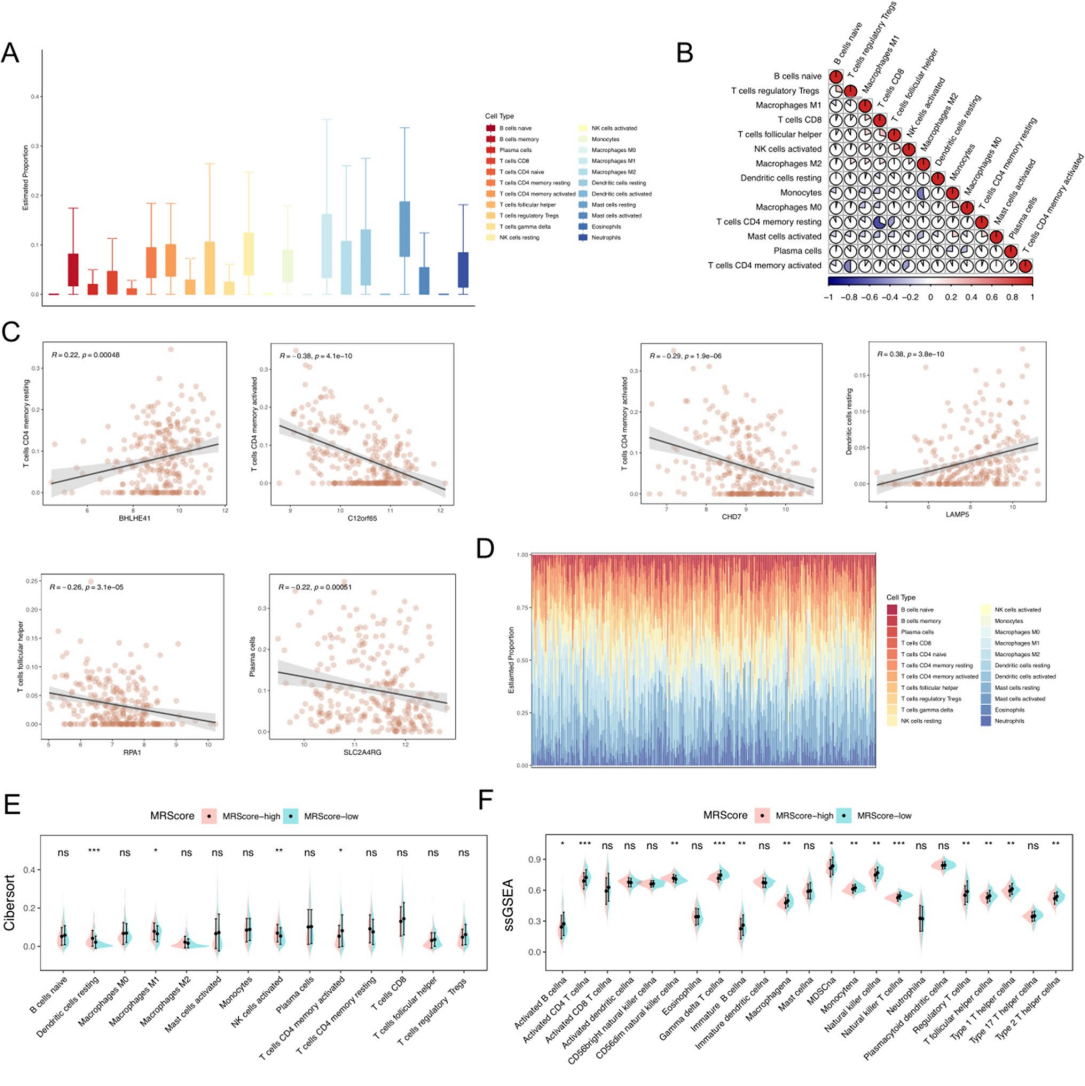
Immune cell infiltration analysis of esophageal cancer prognostic risk model based on m^1^A and m^7^G modification-related genes. **A** Box plot visualization of CIBERSORT to analyze expression of immune cell- associated genes in the constructed esophageal cancer prognostic risk model. **B** Correlation heat map to assess the correlation of expression of immune cell- associated genes in the ESCC patient population. **C** Risk score and several key genes were linearly correlated with the expression of immune cell- associated genes. **D** Stack plot visualization to analyze the expression of immune cell-associated genes in the constructed esophageal cancer prognostic risk model. **E**, **F** Violin plots depict immune infiltration analysis of CIBERSORT and ssGSEA

Analysis of the risk model composed of m^1^A and m^7^G modification-related genes for correlations to immune infiltration in the tumor microenvironment utilizing the CIBERSORT algorithm in ESCC patients showed that naive B cells, M2 macrophages, and CD8^+^ T cells were significantly enriched (Fig. 5A). There was no significant correlation among different type of immune cells (Fig. 5B). Comparison of the linear correlations between the infiltration of immune cells and key gene showed that the *BHLHE41* expression was proportional to the abundance score of resting memory CD4^+^ T cells, moreover, *C12orf65* and *CHD7* expression negatively correlated with the abundance score of activated memory CD4^+^ T cells. *LAMP5* expression showed a significant positive correlation with the expression of resting dendritic CD4^+^ T cells, while follicular helper T cells and plasma cells showed a negative correlation with the expression of *RPA1* and *SLC2A4RG* (Fig. 5C).

The abundance of immune cells in each ESCC patient (Fig. 5D) and immune cell infiltration and the distribution of immune infiltration pathways in the MRScore-low and MRScore-high groups using the CIBERSORT and ssGSEA algorithm showed that compared to the MRScore-low group, patients in the MRScore-high group were correlated to less infiltration of most types of immune cells (Fig. 5E, F).

**Fig. 6:**
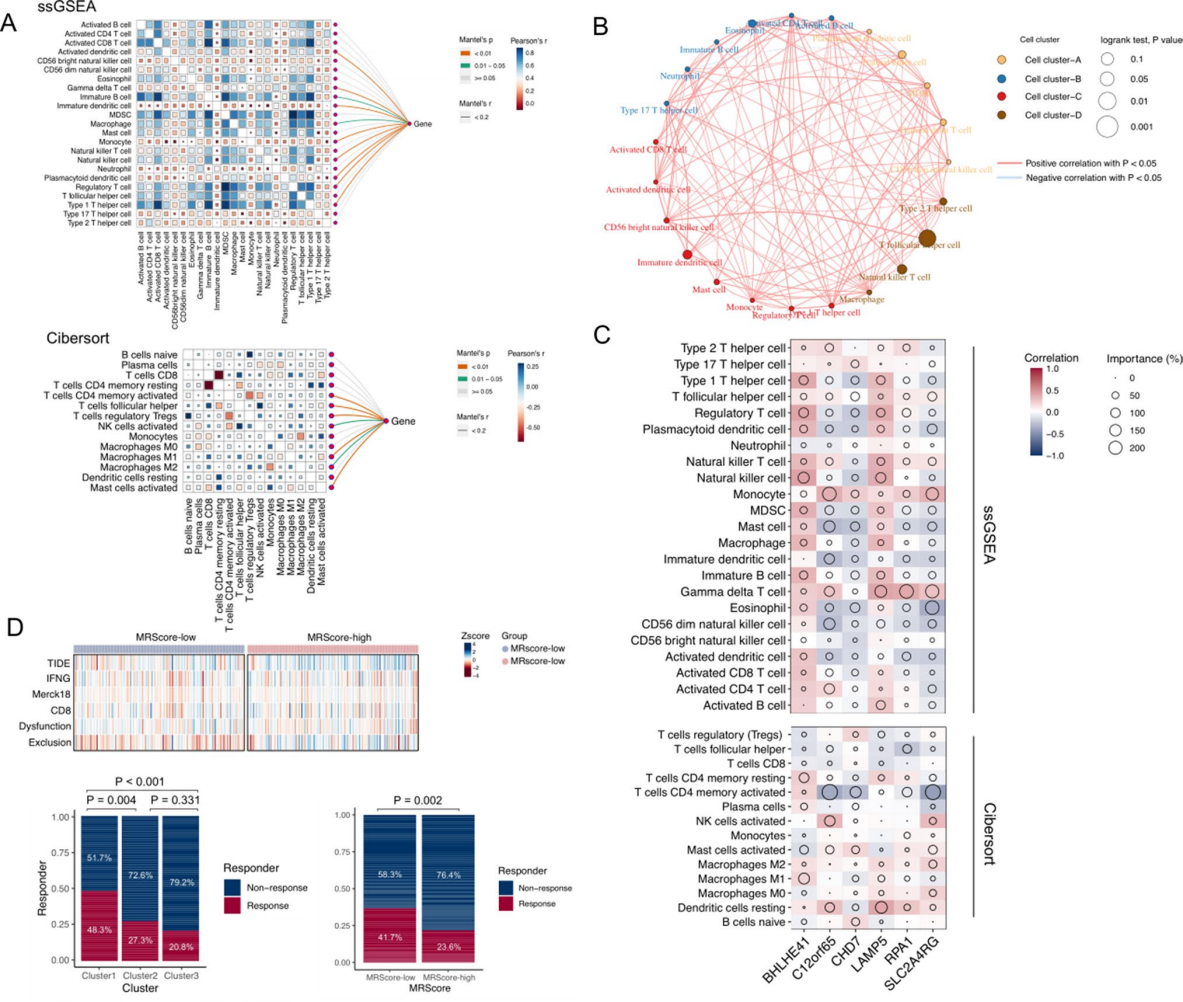
Correlation of RNA methylation model and immune cell infiltration analysis. **A** Correlation of gene expression levels between different immune cells of ssGSEA and CIBERSORT. **B** Spherical plots of expression of immune cell- associated genes in different cell clusters. **C** Correlation heatmap of CIBERSORT and ssGSEA immune infiltration and expression of 6 key genes in the RNA methylation model. **D** Histogram and heatmap of expression of TIDE score in MRScore-high and MRScore-low groups of methylation-related risk model

Figure 6A specifically shows the link between the 6 selected genes and immune cell infiltration. Analysis of correlations between various cell clusters and immune cell infiltration of ssGSEA further showed a significant positive correlation between cell cluster D and NK-T, and Th2 cells, and a negative correlation between cell cluster B and neutrophils (Fig. 6B). Correlations between expression levels of 6 genes and various immune cells are shown in Fig. 6C. Figure 6E illustrates the correlations between the scores of TIDE, IFNG, Merck18, and CD8 expression and the grouping of methylation alterations with high and low MRScore. The therapeutic responsiveness of the MRScore-high group was worse than that of the MRScore-low group (Fig. 6D). Correspondingly, cluster 1 was more responsive than clusters 2 and 3.

**Fig. 7:**
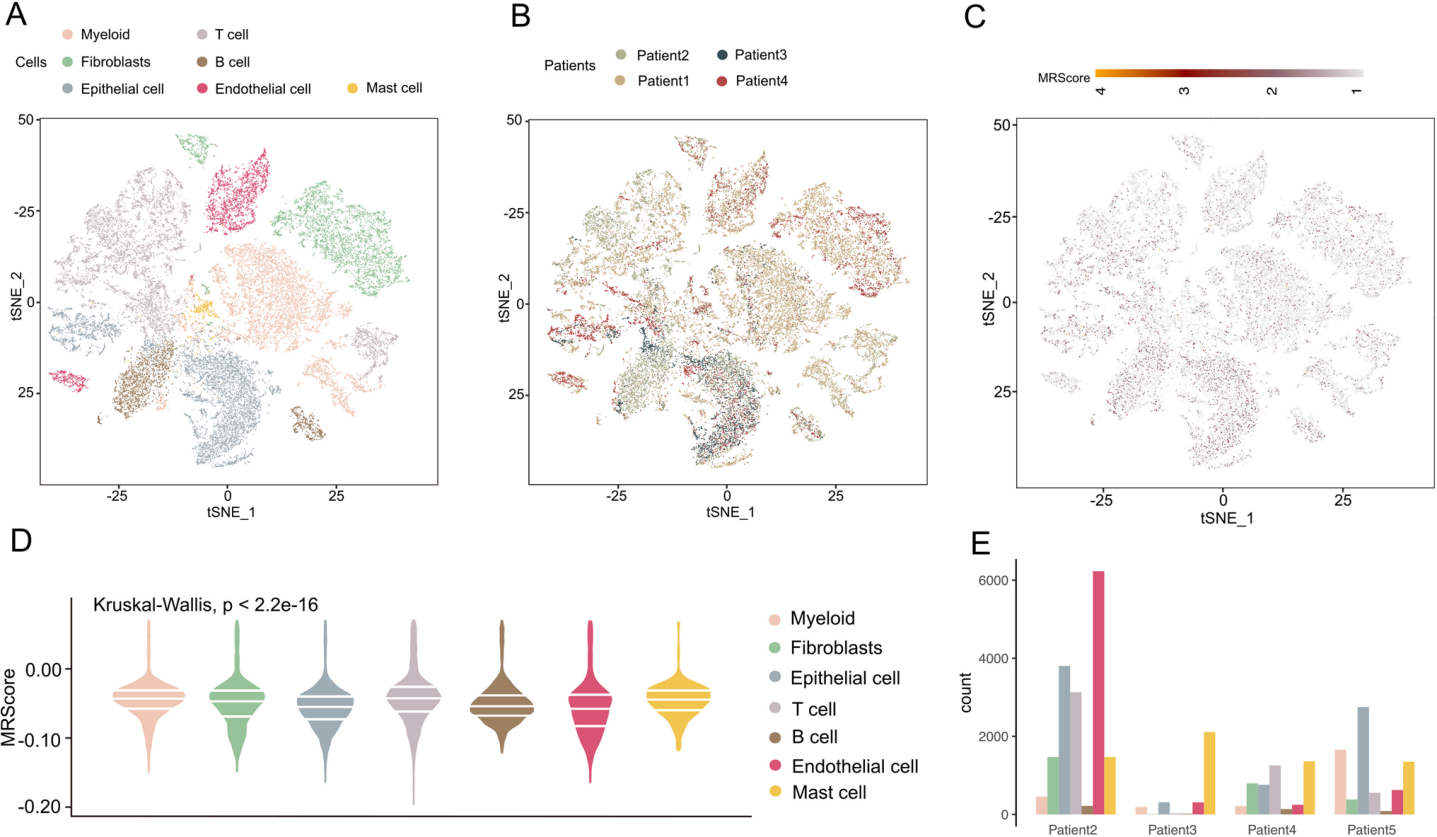
scRNA-seq analysis. **A** t-SNE plot of immune cell class distribution in ESCC patients. **B** Graph demonstrating the four ESCC patients' regional descending fractionation effect.** C** t-SNE plot showing the distribution of the MRScore. **D** Boxplot of the MRScore levels in various cells. **E** Stacked graph depicting the immune cell type composition of four ESCC patients

In addition, we analyzed scRNA-seq data derived from four ESCC patients, thereby facilitating a comprehensive exploration of RNA methylation-associated immune infiltration and chemotherapeutic susceptibility. Several classes of immune cells were successfully classified into specific regional types (Fig. 7A), and the impact of regional descending fractionation in the four patients was demonstrated through t-SNE plots (Fig. 7B). As shown in Fig. 7C, we annotated the MRScore with descending binned clusters. Kruskal–Wallis test shows significant variations in the distribution of MRScore in different cells (Fig. 7D). In Fig. 7E, we precisely analyzed the cell type composition of the four ESCC patients using stacked plots, and the results showed that T cells, Myeloid cells, and Fibroblasts were more enriched compared to other.

### Drug sensitivity analysis and functional evaluation related to the prognostic risk model

**Fig. 8:**
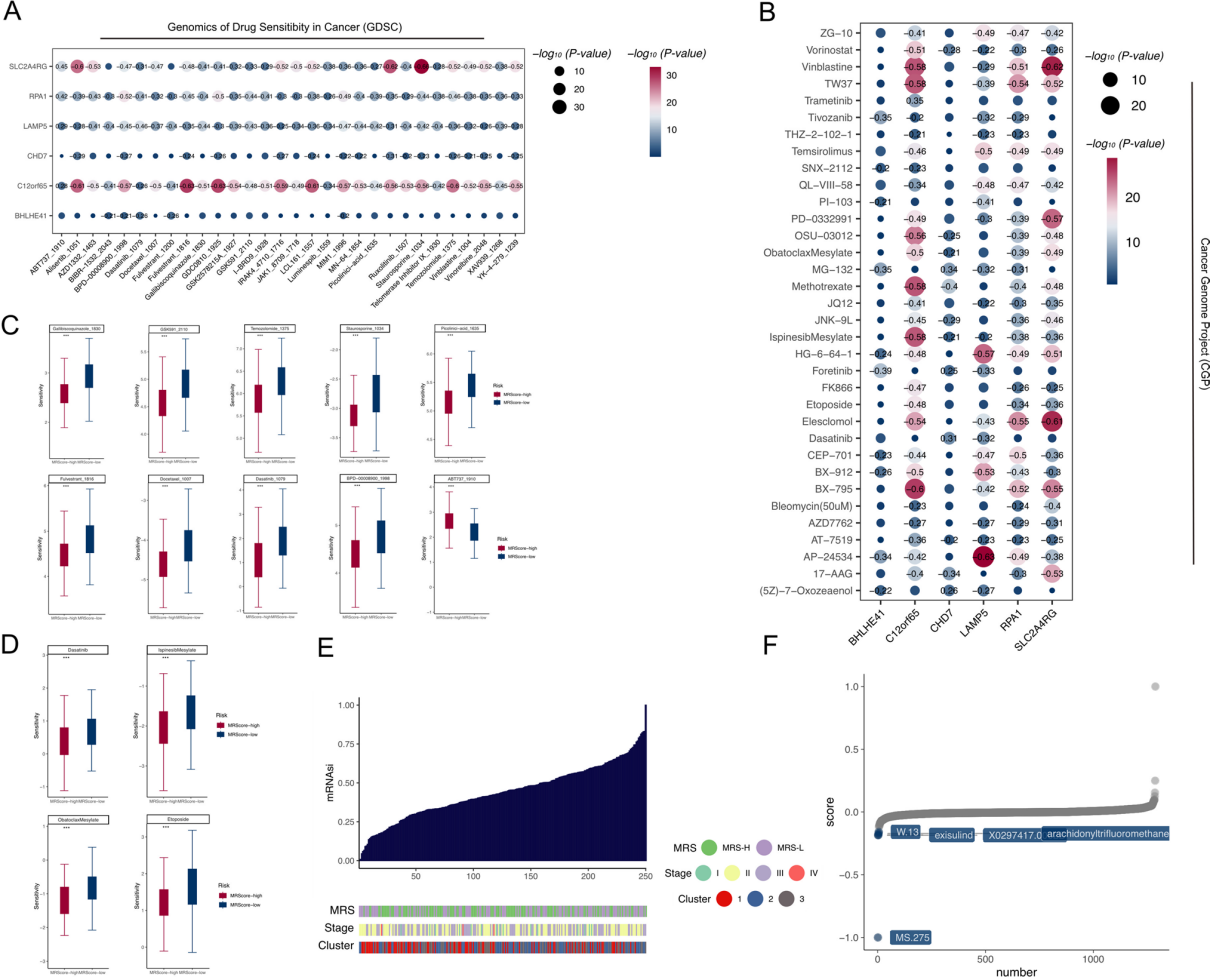
Treatment sensitivity evaluation of prognostic risk model in ESCC. **A** Correlation analysis of chemotherapeutic drug sensitivity from the GDSC database with 6 key genes. **B** Correlation analysis of drug sensitivity from the CGP database with the 6 key genes. **C**, **D** Box plot of the difference in sensitivity of multiple chemotherapeutic drugs in MRScore-low and MRScore-high groups in the prognostic risk model.** E** mRNAsi scores based on transcriptome expression in ESCC patients. **F** CMap analysis of the mRNAsi molecular signature

Results of the analysis of correlations between the RNA methylation risk model and predicted score (IC50) obtained from the GDSC database (Fig. 8A) and CGP database (Fig. 8B) predicted drug sensitivity. We further predicted the difference in treatment sensitivity between the MRScore-high and MRScore-low groups for several classes of drugs, including Docetaxel, Temozolomide, Fulvestrant, Dasatinib, Etoposide and et al. Results suggested that the MRScore-high group was significantly more sensitive than the MRScore-low group (Fig. 8C, D). The degree of dedifferentiation of oncogenic stem cells was calculated from the stemness index (mRNAsi) based on transcriptome expression in ESCC patients (Fig. 8E). Five potentially therapeutic molecules with the lowest CMap scores were identified from the CMap drug database using CMap analysis of the mRNAsi molecular signatures. These five molecules are potential oncology therapeutics for reversing oncogenic stem cell dedifferentiation. (Fig. 8F).

## Discussion

Although the incidence and mortality rates of esophageal cancer have been significantly reduced recent years, the OS of esophageal cancer patients after surgery remains low and the prognosis is poor [46]. Molecular and systems studies on the developmental mechanism of esophageal cancer can provide new, precise, and targeted therapeutic strategies for the treatment of esophageal cancer to improve prognosis and OS rates and are, therefore, research hotspot in the field of esophageal cancer [47, 48]. The function of their related enzymes or proteins in the development of esophageal cancer has also been widely investigated. Studies have shown that the up-regulated expression of methylation-related genes can promote tumor proliferation, migration, and invasion [49–51]. However, studies on the role of m^1^A- and m^7^G- methylation- related genes in esophageal cancer have not been reported, and their mechanisms of action in esophageal cancer remain unclear. In this study, 23 genes related to m^1^A and m^7^G modification were identified based on previously validated and reliable studies, and their associated biological pathways and pharmacological sensitivities were systematically characterized. The m^1^A and m^7^G genes could conceivably orchestrate immune responses, potentially dictating the degree of immune cell infiltration and subsequent immunological activities in pathological scenarios.

Our comprehensive analysis identified an intricate association between DEGs and multiple dimensions of tumor advancement. These include metabolic alterations, cellular processes, microenvironmental shifts, and genetic information processing. A correlation heatmap was employed to unveil the functional connectivity embedded within 23 distinct m^1^A and m^7^G modification-associated genes, all of which have potential implications in prognostic stratification for ESCC patients. Our meticulously developed risk model, premised on this genetic composition, presented a robust tool for patient prognosis stratification, and uncovered significant correlations with immune infiltration and drug resistance phenomena.

The immune microenvironment of esophageal cancer is closely related to tumorigenesis, progression, and prognosis, and the immune cell infiltration characteristics regulate the response to ICIs [52]. Formerly, it was difficult to determine the composition ratio of infiltrating immune cells in tumor tissues by immunohistochemistry and flow cytometry, so the immune panorama of esophageal cancer tissues was hard to show. Meanwhile, tumor mutation burden and PD-L1 expression are not able to accurately predict the efficacy of immunotherapy yet [53, 54]. Researchers are committed to exploring easier ways to screen potential populations who will benefit from immunotherapy, which has made a splash in the treatment of ESCC [55]. The sextet of pivotal genes (BHLHE41, C12orf65, CHD7, LAMP5, RPA1, SLC2A4RG) serve as fundamental players in prognosticating ESCC, each of them has been highlighted in preceding research concerning diverse types of malignancies. Dysregulation of BHLHE41, a gene implicated in tumor progression, has been substantiated within the context of breast and colorectal cancers [56, 57]. Additionally, mutations in C12orf65 have been postulated to engender carcinogenic consequences through perturbations in mitochondrial functionality [58]. Equally notable are CHD7 mutations, previously identified within neuroblastoma and colorectal cancer narratives [59]. Within distinct neoplastic entities, modulations of LAMP5's lysosomal operation bear the potential to affect cancer cellular dynamics [60]. Furthermore, the alteration in the gene RPA1, integral to DNA repair machineries, has been correlated with breast and ovarian cancers [61]. Lastly, SLC2A4RG, with its involvement in glucose metabolism, is connected to deviations in metabolic activities instrumental in tumor progression [62]. Nevertheless, their exact roles in ESCC remain notably elusive. This indeed underscores a crucial lacuna in our current knowledge and underscores the pressing need for additional research to determine their specific function and prognostic utility in ESCC.

Intricately entwined in the m^1^A and m^7^G methylation alterations, these genes potentially modulate immune cell infiltration and chemotherapeutic susceptibility, thereby shaping patient survival outcomes and therapeutic responsiveness in the complex landscape of ESCC. We explored the differences in immune cell infiltration and tumor microenvironment scoring between MRScore-high and MRScore-low groups of patients and found that methylation transcriptomic markers were closely associated with the immune infiltration of esophageal cancer tumor cells and the expression level of immune cells. We used multiple methods to further investigate the predictive role of the risk model on the prognosis of ESCC patients. Kaplan–Meier curves showed that the survival of patients in the high-risk group was significantly worse than that of low-risk patients, and the accuracy of the model was further validated by ROC curves, nomograms, and calibration curves. The risk model also correlated with the staging classification of ESCC and had a better predictive effect on the prognosis of patients in different clinical subgroups. The distribution and regional positions of the three stable subtypes in the CDF curve indicated that the clustering analysis model has a high predictive performance. Our predictive model, accentuating m^1^A and m^7^G methylation deviations, confers superior prognostic accuracy for ESCC patients juxtaposed to traditional frameworks, while concurrently delivering profound revelations into immune cell infiltration and chemotherapeutic susceptibility.

This study also has some limitations. Firstly, as a retrospective analysis, there are limitations in data acquisition. Second, the study design was biased in terms of variable selection, with a small sample size of the ESCC cohort in public databases. Also, the GEO database lacks complete treatment records, such as the choice of chemotherapy regimen or information on targeted therapies. Furthermore, some factors of laboratory tests, such as tumor markers, are also important for the survival of cancer patients, and the omission of these underlying factors might affect the prognostic assessment results of methylation prediction models for ESCC patients. It is hoped that future related studies will incorporate these important factors to improve the prognostic value of molecular markers of transcriptomics associated with m^1^A and m^7^G modification.

## Conclusion

Transcriptomic prognostic markers based on m^1^A and m^7^G modification for the prognostic evaluation of ESCC showed a robust correlation with the expression of immune infiltration cells and immune pathways in ESCC patients. Scores from the risk model based on the composition of genes associated with m^1^A and m^7^G modification were significantly correlated with the therapeutic sensitivity of multiple chemotherapeutic agents. This suggests an important clinical value of our constructed prognostic markers based on m^1^A and m^7^G modification, which may promote the future development of accurate diagnosis and individualized medicine for ESCC patients.

## Supplementary Information

**Additional file 1: Figure S1.** Flow chart of this study. **Figure S2.** Removal of batch effects, consensus clustering and variance analysis. (A) PCA plots before and after removal of batch effects. (B) Distribution of the 23 m1A and m7G modification-related key genes on human chromosomes. (C) Correlation heat map analysis of functional connectivity between the 23 m1A and m7G modification-related genes and expression content correlation. (D) Display graphs of consensus clustering analysis (K value of consensus matrix is set at 2,4,5,6). (E) Hosmer-Leme test showed the prognostic model had good goodness of fit.

**Additional file 2:** **Table S1.** Summary of 23m1A and m7G regulators. **Table S2.** The characteristics of TCGA, GSE53625 and SYSUCC cohorts. **Table S3.** The activation states of GO and KEGG terms between three ESCC clusters. **Table S4.** GSEA of significant KEGG terms between three ESCC clusters. **Table S5.** Summary of 6 key prognostic genes.

## Data Availability

All datasets generated for this study are included in the article material, including TCGA-ESCC database and GEO dataset (https://www.ncbi.nlm.nih.gov/gds/): GSE53625, GSE188900.
