# Supplementary material for: M1A and m7G modification-related genes are potential biomarkers for survival prognosis and for deciphering the tumor immune microenvironment in esophageal squamous cell carcinoma
Source: Discov Oncol. 2023 Jun 14;14:99. doi: 10.1007/s12672-023-00710-6 (PMC10267068; doi:10.1007/s12672-023-00710-6)
Supplement: Supplementary file 1 — Additional file 1: Figure S1. Flow chart of this study. Figure S2. Removal of batch effects, consensus clustering and variance analysis. (A) PCA plots before and after removal of batch effects. (B) Distribution of the 23 m1A and m7G modification-related key genes on human chromosomes. (C) Correlation heat map analysis of functional connectivity between the 23 m1A and m7G modification-related genes and expression content correlation. (D) Display graphs of consensus clustering analysis (K value of consensus matrix is set at 2,4,5,6). (E) Hosmer-Leme test showed the prognostic model had good goodness of fit. [file 12672_2023_710_MOESM1_ESM.docx]

M^1^A and m^7^G modification-related genes are potential biomarkers for survival prognosis and for deciphering the tumor immune microenvironment in esophageal squamous cell carcinoma

Ruixi Wang^1,2†^, Xingyuan Cheng^1,2†^, Dongmei Chi^1,3†^, Shiliang Liu^1,2^, Qiaoqiao Li^1,2^, Baoqing Chen^1,2*^ and Mian Xi^1,2*^

^1^State Key Laboratory of Oncology in South China, Collaborative Innovation Centre for Cancer Medicine, Guangdong Esophageal Cancer Institute, Guangzhou, China

^2^Department of Radiation Oncology, Sun Yat-Sen University Cancer Center, No. 651 Dongfeng East Road, Guangzhou 510060, China

^3^Department of Anesthesiology, Sun Yat-Sen University Cancer Center, No. 651 Dongfeng East Road, Guangzhou 510060, China

^†^Equal contribution as co-first authors

***Correspondence:**

Dr. Mian Xi, Department of Radiation Oncology, Sun Yat-sen University Cancer Center, No.651 Dongfeng East Road, Guangzhou 510060, China. Tel: +86-20-87341614, Fax: +86-20-87343492, Email: [ximian@sysucc.org.cn](mailto:ximian@sysucc.org.cn)

Dr. Baoqing Chen, Department of Radiation Oncology, Sun Yat-sen University Cancer Center, No.651 Dongfeng East Road, Guangzhou 510060, China. Tel: +86-20-87341614, Fax: +86-20-87343492, Email: chenbq@sysucc.org.cn

# Supplementary Figure 1. Flow chart of this study.


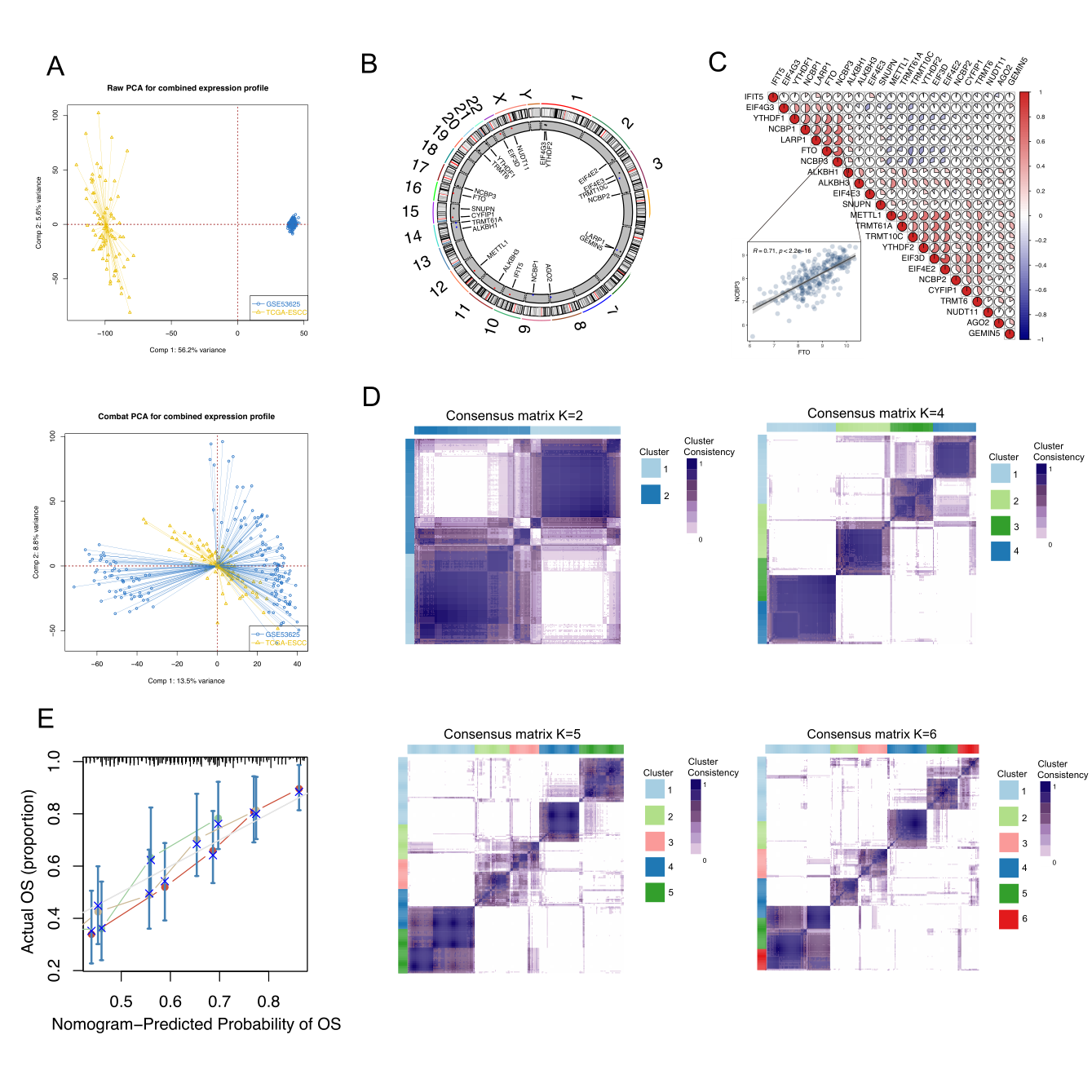


**Supplementary Figure 2.** Removal of batch effects, consensus clustering and variance analysis. **(A)** PCA plots before and after removal of batch effects. **(B)** Distribution of the 23 m^1^A and m^7^G modification-related key genes on human chromosomes. **(C)** Correlation heat map analysis of functional connectivity between the 23 m^1^A and m^7^G modification-related genes and expression content correlation. **(D)** Display graphs of consensus clustering analysis (K value of consensus matrix is set at 2,4,5,6). **(E)** Hosmer-Leme test showed the prognostic model had good goodness of fit.
